# 2-Cyclo­heptyl­idene-*N*-phenyl­hydrazine­carbo­thio­amide

**DOI:** 10.1107/S1600536814003948

**Published:** 2014-02-26

**Authors:** Mehmet Akkurt, Shaaban K. Mohamed, Joel T. Mague, Alaa A. Hassan, Mustafa R. Albayati

**Affiliations:** a Department of Physics, Faculty of Sciences, Erciyes University, 38039 Kayseri, Turkey; b Chemistry and Environmental Division, Manchester Metropolitan University, Manchester M1 5GD, England; c Chemistry Department, Faculty of Science, Minia University, 61519 El-Minia, Egypt; d Department of Chemistry, Tulane University, New Orleans, LA 70118, USA; e Kirkuk University, College of Science, Department of Chemistry, Kirkuk, Iraq

## Abstract

In the title compound, C_14_H_19_N_3_S, the seven-membered cyclo­heptane ring adopts a chair conformation. An intra­molecular N—H⋯N hydrogen bond [graph-set motif *S*(5)] is present in the N—N—C—N chain between the ring systems. An intra­molecular C—H⋯S contact also occurs. In the crystal, pairs of mol­ecules form centrosymmetric dimers through N—H⋯S hydrogen bonds [graph-set *R*_2_^2^(8)]. These dimers are connected by C—H⋯S inter­actions with an *R*_2_^2^(14) motif.

## Related literature   

For the coordination chemistry of thio­semicarbazones, see: Gingras *et al.* (1961 ▶); Ali & Livingstone (1974 ▶); Lobana *et al.* (2009 ▶). For general biological properties of thio­semicarbazone scaffold compounds, see: Hu *et al.* (2006 ▶); Du *et al.* (2002 ▶); Lovejoy & Richardson (2002 ▶). For hydrogen-bond motifs, see: Bernstein *et al.* (1995 ▶). For ring-puckering parameters, see: Cremer & Pople (1975 ▶).
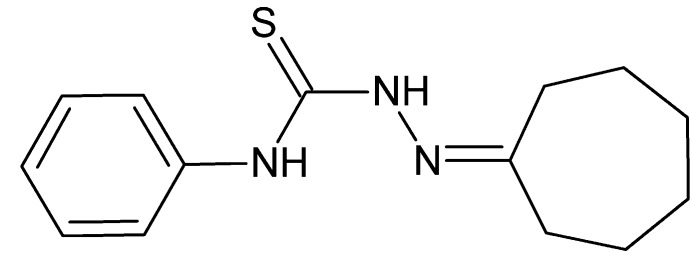


## Experimental   

### 

#### Crystal data  

C_14_H_19_N_3_S
*M* *_r_* = 261.39
Monoclinic, 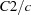

*a* = 22.1371 (4) Å
*b* = 6.1079 (1) Å
*c* = 22.0796 (5) Å
β = 113.219 (2)°
*V* = 2743.61 (10) Å^3^
*Z* = 8
Cu *K*α radiation
μ = 1.97 mm^−1^
*T* = 100 K
0.20 × 0.08 × 0.04 mm

#### Data collection  

Bruker D8 VENTURE PHOTON 100 CMOS diffractometer
Absorption correction: multi-scan (*SADABS*; Bruker, 2013 ▶) *T*_min_ = 0.83, *T*_max_ = 0.93
11263 measured reflections
2693 independent reflections
2460 reflections with *I* > 2σ(*I*)
*R*_int_ = 0.023

#### Refinement  

*R*[*F* ^2^ > 2σ(*F* ^2^)] = 0.030
*wR*(*F* ^2^) = 0.077
*S* = 1.07
2693 reflections
171 parameters
H atoms treated by a mixture of independent and constrained refinement
Δρ_max_ = 0.24 e Å^−3^
Δρ_min_ = −0.24 e Å^−3^

### 

Data collection: *APEX2* (Bruker, 2013 ▶); cell refinement: *SAINT* (Bruker, 2013 ▶); data reduction: *SAINT*; program(s) used to solve structure: *SHELXS2013* (Sheldrick, 2008 ▶); program(s) used to refine structure: *SHELXL2013* (Sheldrick, 2008 ▶); molecular graphics: *ORTEP-3 for Windows* (Farrugia, 2012 ▶); software used to prepare material for publication: *WinGX* (Farrugia, 2012 ▶) and *PLATON* (Spek, 2009 ▶).

## Supplementary Material

Crystal structure: contains datablock(s) global, I. DOI: 10.1107/S1600536814003948/bt6964sup1.cif

Structure factors: contains datablock(s) I. DOI: 10.1107/S1600536814003948/bt6964Isup2.hkl

Supporting information file. DOI: 10.1107/S1600536814003948/bt6964Isup3.cml

CCDC reference: 988001

Additional supporting information:  crystallographic information; 3D view; checkCIF report

## Figures and Tables

**Table 1:** Hydrogen-bond geometry (Å, °)

*D*—H⋯*A*	*D*—H	H⋯*A*	*D*⋯*A*	*D*—H⋯*A*
N1—H1*N*⋯N3	0.857 (18)	2.052 (18)	2.5599 (16)	117.2 (16)
N2—H2*N*⋯S1^i^	0.858 (19)	2.830 (19)	3.6790 (13)	170.5 (15)
C2—H2⋯S1	0.95	2.60	3.2660 (15)	128
C9—H9*A*⋯S1^i^	0.99	2.69	3.3141 (13)	121

Symmetry code: (i) 
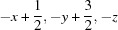
.

**Fig. 1.**
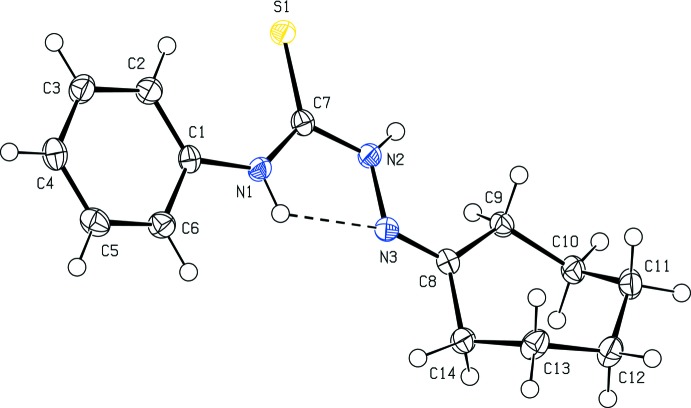
The molecular structure of the title compound with 50% probability displacement ellipsoids.

**Fig. 2.**
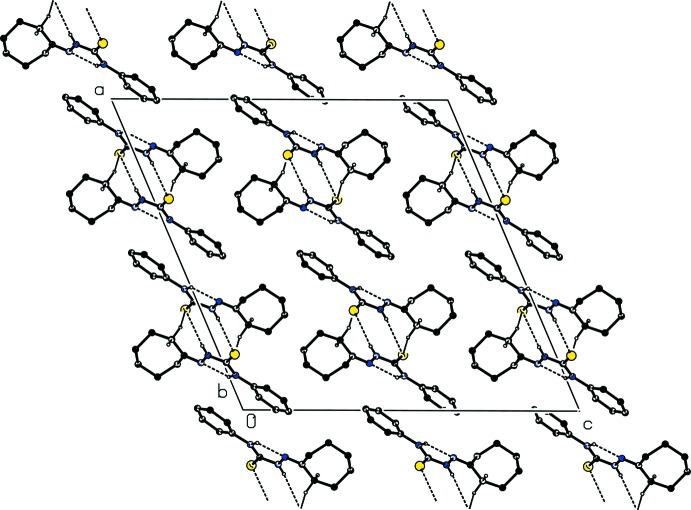
View of the centrosymmetric R22(8) dimers of the title compound viewed down b-axis. H atoms not involved in hydrogen bonding have been omitted for clarity.

## supplementary crystallographic information

### 1. Comment

Thiosemicarbazones constitute an important class of N, S-donor ligands, and their coordination chemistry was initially explored in the early sixties (Gingras *et al.*, 1961; Ali & Livingstone, 1974; Lobana *et al.*, 2009). On other hand, depending on the parent aldehyde or ketone, the corresponding thiosemicarbazone scaffolds have been evaluated over the last 50 years as anti-viral, anti-bacterial and anti-cancer therapeutic agents (Hu *et al.*, 2006; Du *et al.*, 2002; Lovejoy & Richardson, 2002). Based on these facts and following our study of cyclization reactions of thiosemicarbazides we report the synthesis and crystal structure of the title compound.

In this compound (Fig. 1), the cycloheptane ring (C8–C14) adopts a chair conformation [puckering parameters (Cremer & Pople, 1975) are Q(2) = 0.4493 (14) Å, φ(2) = 126.68 (18)° and Q(3) = 0.6604 (14) Å, φ(3) = 102.94 (12)°]. The C1–N1–C7–S1, C1–N1–C7–N2 and N1–C7–N2–N3 torsion angles are 3.8 (2), -176.94 (12) and -2.53 (16) °, respectively.

The molecular conformation of the title compound is stabilized by a cyclic intramolecular N1—H1N···N3 hydrogen bond, forming a graph set *S*(5) (Table 1; Bernstein *et al.*, 1995).

In the crystal, pairs of molecules form centrosymmetric dimers through intermolecular N—H···S hydrogen bonds [graph-set *R*_2_^2^(8)]. These dimers are also connected by C—H···S interactions with an *R*_2_^2^(14) motif.

### 2. Experimental

A solution of 1 mmol (112 mg) of cycloheptanone in 5 ml DMSO was added dropwise to a solution of 1 mmol (167 mg) of *N*-phenylhydrazinecarbothioamide in 5 ml of DMSO. The reaction mixture was stirred for 2 h at ambient temperature and then left to stand overnight. The resulting mixture was poured into 250 ml of ice/water to give a white precipitate. The crude product was filtered off, washed with cold ethanol and recystallized from ethanol to furnish colourless crystals suitable for X-ray diffraction. *M*.p. 379 K.

### 3. Refinement

All H atoms were found in a difference map. All C-bonded H-atoms were positioned geometrically and refined using a riding model [C—H = 0.95 (aromatic H) and 0.99 Å (methylene H)], with *U*_iso_(H) = 1.2 *U*_iso_(C). The N-bonded H-atoms were refined freely.

### Crystal data

**Table:**

C_14_H_19_N_3_S	*F*(000) = 1120
*M**_r_* = 261.39	*D*_x_ = 1.266 Mg m^−^^3^
Monoclinic, *C*2/*c*	Cu *K*α radiation, λ = 1.54178 Å
Hall symbol: -C 2yc	Cell parameters from 8726 reflections
*a* = 22.1371 (4) Å	θ = 4.4–72.4°
*b* = 6.1079 (1) Å	µ = 1.97 mm^−^^1^
*c* = 22.0796 (5) Å	*T* = 100 K
β = 113.219 (2)°	Parallelepiped, colourless
*V* = 2743.61 (10) Å^3^	0.20 × 0.08 × 0.04 mm
*Z* = 8	

### Data collection

**Table:**

Bruker D8 VENTURE PHOTON 100 CMOS diffractometer	2693 independent reflections
Radiation source: INCOATEC IµS micro–focus source	2460 reflections with *I* > 2σ(*I*)
Mirror monochromator	*R*_int_ = 0.023
Detector resolution: 10.4167 pixels mm^-1^	θ_max_ = 72.4°, θ_min_ = 4.4°
ω scans	*h* = −25→27
Absorption correction: multi-scan (*SADABS*; Bruker, 2013)	*k* = −7→7
*T*_min_ = 0.83, *T*_max_ = 0.93	*l* = −27→27
11263 measured reflections	

### Refinement

**Table:**

Refinement on *F*^2^	0 restraints
Least-squares matrix: full	Hydrogen site location: mixed
*R*[*F*^2^ > 2σ(*F*^2^)] = 0.030	H atoms treated by a mixture of independent and constrained refinement
*wR*(*F*^2^) = 0.077	*w* = 1/[σ^2^(*F*_o_^2^) + (0.0368*P*)^2^ + 2.2793*P*] where *P* = (*F*_o_^2^ + 2*F*_c_^2^)/3
*S* = 1.07	(Δ/σ)_max_ < 0.001
2693 reflections	Δρ_max_ = 0.24 e Å^−^^3^
171 parameters	Δρ_min_ = −0.24 e Å^−^^3^

### Special details

**Table:**

Geometry. Bond distances, angles *etc*. have been calculated using the rounded fractional coordinates. All su's are estimated from the variances of the (full) variance-covariance matrix. The cell e.s.d.'s are taken into account in the estimation of distances, angles and torsion angles
Refinement. Refinement on *F*^2^ for ALL reflections except those flagged by the user for potential systematic errors. Weighted *R*-factors *wR* and all goodnesses of fit *S* are based on *F*^2^, conventional *R*-factors *R* are based on *F*, with *F* set to zero for negative *F*^2^. The observed criterion of *F*^2^ > σ(*F*^2^) is used only for calculating -*R*-factor-obs *etc*. and is not relevant to the choice of reflections for refinement. *R*-factors based on *F*^2^ are statistically about twice as large as those based on *F*, and *R*-factors based on ALL data will be even larger.

### Fractional atomic coordinates and isotropic or equivalent isotropic displacement parameters (Å^2^)

**Table:**

	*x*	*y*	*z*	*U*_iso_*/*U*_eq_	
S1	0.17859 (2)	0.78279 (5)	0.04498 (2)	0.0211 (1)	
N1	0.11142 (5)	0.39799 (19)	0.02178 (5)	0.0198 (3)	
N2	0.17477 (5)	0.46222 (18)	−0.03562 (5)	0.0198 (3)	
N3	0.15155 (5)	0.26265 (17)	−0.06567 (5)	0.0194 (3)	
C1	0.07507 (6)	0.4095 (2)	0.06186 (6)	0.0184 (3)	
C2	0.07037 (6)	0.5909 (2)	0.09770 (6)	0.0238 (4)	
C3	0.03058 (6)	0.5801 (2)	0.13315 (7)	0.0255 (4)	
C4	−0.00440 (6)	0.3922 (2)	0.13341 (6)	0.0246 (4)	
C5	0.00110 (7)	0.2115 (2)	0.09818 (7)	0.0260 (4)	
C6	0.04086 (6)	0.2191 (2)	0.06301 (6)	0.0226 (4)	
C7	0.15252 (6)	0.5377 (2)	0.01003 (6)	0.0187 (3)	
C8	0.17441 (6)	0.1871 (2)	−0.10668 (6)	0.0186 (3)	
C9	0.22606 (6)	0.3024 (2)	−0.12320 (6)	0.0201 (3)	
C10	0.24745 (6)	0.1938 (2)	−0.17382 (6)	0.0221 (4)	
C11	0.19353 (7)	0.1805 (2)	−0.24352 (6)	0.0254 (4)	
C12	0.14582 (7)	−0.0109 (2)	−0.25458 (6)	0.0261 (4)	
C13	0.10499 (6)	−0.0088 (2)	−0.21264 (6)	0.0259 (4)	
C14	0.14621 (6)	−0.0277 (2)	−0.13788 (6)	0.0217 (4)	
H1N	0.1062 (8)	0.280 (3)	−0.0007 (8)	0.023 (4)*	
H2	0.09410	0.72090	0.09800	0.0290*	
H2N	0.2057 (8)	0.535 (3)	−0.0405 (8)	0.025 (4)*	
H3	0.02730	0.70410	0.15770	0.0310*	
H4	−0.03180	0.38760	0.15740	0.0300*	
H5	−0.02250	0.08150	0.09810	0.0310*	
H6	0.04480	0.09350	0.03950	0.0270*	
H9A	0.26550	0.32090	−0.08190	0.0240*	
H9B	0.20950	0.45070	−0.13950	0.0240*	
H10A	0.26290	0.04360	−0.15860	0.0270*	
H10B	0.28510	0.27640	−0.17570	0.0270*	
H11A	0.16810	0.31870	−0.25300	0.0300*	
H11B	0.21460	0.16830	−0.27540	0.0300*	
H12A	0.11540	−0.01210	−0.30160	0.0310*	
H12B	0.17130	−0.14880	−0.24570	0.0310*	
H13A	0.07340	−0.13190	−0.22650	0.0310*	
H13B	0.07930	0.12880	−0.22130	0.0310*	
H14A	0.11820	−0.08710	−0.11620	0.0260*	
H14B	0.18260	−0.13250	−0.13040	0.0260*	

### Atomic displacement parameters (Å^2^)

**Table:**

	*U*^11^	*U*^22^	*U*^33^	*U*^12^	*U*^13^	*U*^23^
S1	0.0234 (2)	0.0197 (2)	0.0226 (2)	−0.0056 (1)	0.0116 (1)	−0.0035 (1)
N1	0.0235 (5)	0.0191 (6)	0.0189 (5)	−0.0053 (4)	0.0106 (4)	−0.0042 (4)
N2	0.0225 (5)	0.0195 (6)	0.0209 (5)	−0.0054 (4)	0.0122 (4)	−0.0025 (4)
N3	0.0215 (5)	0.0183 (5)	0.0188 (5)	−0.0021 (4)	0.0084 (4)	−0.0008 (4)
C1	0.0151 (5)	0.0240 (7)	0.0148 (5)	−0.0009 (5)	0.0046 (4)	0.0016 (5)
C2	0.0252 (6)	0.0235 (7)	0.0249 (6)	−0.0062 (5)	0.0123 (5)	−0.0032 (5)
C3	0.0280 (7)	0.0277 (7)	0.0242 (6)	−0.0015 (6)	0.0138 (6)	−0.0035 (5)
C4	0.0209 (6)	0.0323 (8)	0.0238 (6)	0.0001 (5)	0.0122 (5)	0.0031 (6)
C5	0.0229 (6)	0.0249 (7)	0.0326 (7)	−0.0038 (5)	0.0136 (6)	0.0026 (6)
C6	0.0216 (6)	0.0222 (7)	0.0247 (6)	−0.0023 (5)	0.0099 (5)	−0.0013 (5)
C7	0.0173 (6)	0.0213 (6)	0.0162 (5)	−0.0001 (5)	0.0051 (5)	0.0018 (5)
C8	0.0180 (6)	0.0203 (6)	0.0179 (6)	0.0000 (5)	0.0074 (5)	0.0024 (5)
C9	0.0214 (6)	0.0199 (6)	0.0202 (6)	−0.0035 (5)	0.0095 (5)	0.0004 (5)
C10	0.0219 (6)	0.0251 (7)	0.0234 (6)	−0.0027 (5)	0.0133 (5)	0.0002 (5)
C11	0.0295 (7)	0.0293 (7)	0.0208 (6)	−0.0028 (6)	0.0136 (5)	0.0005 (5)
C12	0.0267 (6)	0.0314 (8)	0.0215 (6)	−0.0038 (6)	0.0109 (5)	−0.0052 (6)
C13	0.0218 (6)	0.0310 (7)	0.0257 (7)	−0.0057 (5)	0.0103 (5)	−0.0080 (6)
C14	0.0230 (6)	0.0217 (7)	0.0251 (6)	−0.0045 (5)	0.0146 (5)	−0.0025 (5)

### Geometric parameters (Å, º)

**Table:**

S1—C7	1.6788 (13)	C12—C13	1.528 (2)
N1—C1	1.4132 (18)	C13—C14	1.5437 (17)
N1—C7	1.3451 (18)	C2—H2	0.9500
N2—N3	1.3861 (15)	C3—H3	0.9500
N2—C7	1.3648 (17)	C4—H4	0.9500
N3—C8	1.2846 (17)	C5—H5	0.9500
N1—H1N	0.857 (18)	C6—H6	0.9500
N2—H2N	0.858 (19)	C9—H9A	0.9900
C1—C6	1.3935 (18)	C9—H9B	0.9900
C1—C2	1.3891 (18)	C10—H10A	0.9900
C2—C3	1.392 (2)	C10—H10B	0.9900
C3—C4	1.3858 (18)	C11—H11A	0.9900
C4—C5	1.3830 (18)	C11—H11B	0.9900
C5—C6	1.385 (2)	C12—H12A	0.9900
C8—C14	1.4988 (17)	C12—H12B	0.9900
C8—C9	1.5051 (19)	C13—H13A	0.9900
C9—C10	1.5267 (18)	C13—H13B	0.9900
C10—C11	1.5334 (18)	C14—H14A	0.9900
C11—C12	1.529 (2)	C14—H14B	0.9900
			
C1—N1—C7	133.21 (11)	C6—C5—H5	120.00
N3—N2—C7	118.48 (11)	C1—C6—H6	120.00
N2—N3—C8	118.59 (11)	C5—C6—H6	120.00
C7—N1—H1N	111.7 (12)	C8—C9—H9A	108.00
C1—N1—H1N	115.1 (12)	C8—C9—H9B	108.00
C7—N2—H2N	117.1 (12)	C10—C9—H9A	108.00
N3—N2—H2N	124.0 (12)	C10—C9—H9B	108.00
N1—C1—C2	125.84 (12)	H9A—C9—H9B	107.00
C2—C1—C6	119.47 (12)	C9—C10—H10A	109.00
N1—C1—C6	114.68 (11)	C9—C10—H10B	109.00
C1—C2—C3	119.26 (12)	C11—C10—H10A	109.00
C2—C3—C4	121.35 (12)	C11—C10—H10B	109.00
C3—C4—C5	119.04 (13)	H10A—C10—H10B	108.00
C4—C5—C6	120.32 (12)	C10—C11—H11A	109.00
C1—C6—C5	120.54 (12)	C10—C11—H11B	109.00
S1—C7—N2	118.81 (10)	C12—C11—H11A	109.00
N1—C7—N2	113.37 (11)	C12—C11—H11B	109.00
S1—C7—N1	127.82 (10)	H11A—C11—H11B	108.00
N3—C8—C9	123.42 (11)	C11—C12—H12A	108.00
N3—C8—C14	115.47 (12)	C11—C12—H12B	108.00
C9—C8—C14	121.12 (11)	C13—C12—H12A	108.00
C8—C9—C10	117.30 (10)	C13—C12—H12B	108.00
C9—C10—C11	114.48 (12)	H12A—C12—H12B	107.00
C10—C11—C12	114.59 (10)	C12—C13—H13A	109.00
C11—C12—C13	115.67 (11)	C12—C13—H13B	109.00
C12—C13—C14	113.95 (11)	C14—C13—H13A	109.00
C8—C14—C13	112.92 (10)	C14—C13—H13B	109.00
C1—C2—H2	120.00	H13A—C13—H13B	108.00
C3—C2—H2	120.00	C8—C14—H14A	109.00
C2—C3—H3	119.00	C8—C14—H14B	109.00
C4—C3—H3	119.00	C13—C14—H14A	109.00
C3—C4—H4	120.00	C13—C14—H14B	109.00
C5—C4—H4	120.00	H14A—C14—H14B	108.00
C4—C5—H5	120.00		
			
C7—N1—C1—C2	5.0 (2)	C1—C2—C3—C4	0.0 (2)
C7—N1—C1—C6	−176.19 (13)	C2—C3—C4—C5	0.7 (2)
C1—N1—C7—S1	3.8 (2)	C3—C4—C5—C6	−0.2 (2)
C1—N1—C7—N2	−176.94 (12)	C4—C5—C6—C1	−0.9 (2)
C7—N2—N3—C8	−177.23 (12)	N3—C8—C9—C10	−178.99 (12)
N3—N2—C7—S1	−178.15 (9)	C14—C8—C9—C10	1.11 (17)
N3—N2—C7—N1	2.53 (16)	N3—C8—C14—C13	113.26 (13)
N2—N3—C8—C14	−179.13 (10)	C9—C8—C14—C13	−66.84 (16)
N2—N3—C8—C9	0.97 (18)	C8—C9—C10—C11	65.39 (14)
N1—C1—C2—C3	177.64 (12)	C9—C10—C11—C12	−81.85 (14)
C2—C1—C6—C5	1.6 (2)	C10—C11—C12—C13	62.65 (16)
C6—C1—C2—C3	−1.15 (19)	C11—C12—C13—C14	−63.37 (14)
N1—C1—C6—C5	−177.31 (12)	C12—C13—C14—C8	82.43 (14)

### Hydrogen-bond geometry (Å, º)

**Table:**

*D*—H···*A*	*D*—H	H···*A*	*D*···*A*	*D*—H···*A*
N1—H1*N*···N3	0.857 (18)	2.052 (18)	2.5599 (16)	117.2 (16)
N2—H2*N*···S1^i^	0.858 (19)	2.830 (19)	3.6790 (13)	170.5 (15)
C2—H2···S1	0.95	2.60	3.2660 (15)	128
C9—H9*A*···S1^i^	0.99	2.69	3.3141 (13)	121

Symmetry code: (i) −*x*+1/2, −*y*+3/2, −*z*.
